# Routine clinical care data from thirteen cardiac outpatient clinics: design of the Cardiology Centers of the Netherlands (CCN) database

**DOI:** 10.1186/s12872-021-02020-7

**Published:** 2021-06-10

**Authors:** Sophie H. Bots, Klaske R. Siegersma, N. Charlotte Onland-Moret, Folkert W. Asselbergs, G. Aernout Somsen, Igor I. Tulevski, Hester M. den Ruijter, Leonard Hofstra

**Affiliations:** 1 Laboratory of Experimental Cardiology, University Medical Center Utrecht, Utrecht University, Utrecht, The Netherlands; 2 Department of Cardiology, Amsterdam University Medical Centres, location VUmc, Amsterdam, The Netherlands; 3 Julius Center for Health Sciences and Primary Care, University Medical Center Utrecht, Utrecht University, Utrecht, The Netherlands; 4 Department of Cardiology, Division Heart and Lungs, University Medical Center Utrecht, Utrecht University, Utrecht, The Netherlands; 5 Institute of Cardiovascular Science, Faculty of Population Health Sciences, University College London, London, UK; 6 Health Data Research UK and Institute of Health Informatics, University College London, London, UK; 7 Cardiology Centers of the Netherlands, Amsterdam, The Netherlands

**Keywords:** Clinical care data, Cardiovascular care, Prevention, Big data

## Abstract

**Background:**

Despite the increasing availability of clinical data due to the digitalisation of healthcare systems, data often remain inaccessible due to the diversity of data collection systems. In the Netherlands, Cardiology Centers of the Netherlands (CCN) introduced “one-stop shop” diagnostic clinics for patients suspected of cardiac disease by their general practitioner. All CCN clinics use the same data collection system and standardised protocol, creating a large regular care database. This database can be used to describe referral practices, evaluate risk factors for cardiovascular disease (CVD) in important patient subgroups, and develop prediction models for use in daily care.

**Construction and content:**

The current database contains data on all patients who underwent a cardiac workup in one of the 13 CCN clinics between 2007 and February 2018 (n = 109,151, 51.9% women). Data were pseudonymised and contain information on anthropometrics, cardiac symptoms, risk factors, comorbidities, cardiovascular and family history, standard blood laboratory measurements, transthoracic echocardiography, electrocardiography in rest and during exercise, and medication use. Clinical follow-up is based on medical need and consisted of either a repeat visit at CCN (43.8%) or referral for an external procedure in a hospital (16.5%). Passive follow-up via linkage to national mortality registers is available for 95% of the database.

**Utility and discussion:**

The CCN database provides a strong base for research into historically underrepresented patient groups due to the large number of patients and the lack of in- and exclusion criteria. It also enables the development of artificial intelligence-based decision support tools. Its contemporary nature allows for comparison of daily care with the current guidelines and protocols. Missing data is an inherent limitation, as the cardiologist could deviate from standardised protocols when clinically indicated.

**Conclusion:**

The CCN database offers the opportunity to conduct research in a unique population referred from the general practitioner to the cardiologist for diagnostic workup. This, in combination with its large size, the representation of historically underrepresented patient groups and contemporary nature makes it a valuable tool for expanding our knowledge of cardiovascular diseases.

*Trial registration*: Not applicable.

**Supplementary Information:**

The online version contains supplementary material available at 10.1186/s12872-021-02020-7.

## Background

Cardiovascular diseases (CVD) remain an important cause of death and disability worldwide [1, 2]. The digitalisation of the healthcare system has made a wealth of clinical care data available for researchers [3–6]. This provides a unique opportunity for researchers to evaluate pressing topics in cardiovascular medicine. The added value of clinical care data in cardiovascular research is threefold. First, clinical care data better reflect the current real-world situation in healthcare with regard to clinical presentation of disease and representation of patient groups. This is especially relevant for patient groups that have historically been underrepresented in clinical studies such as women [7], the elderly [8] and patients with multimorbidity [9]. CVD in women may be different from CVD in men in several aspects, including the clinical presentation, the effect of traditional risk factors and presence of female-specific risk factors related to pregnancy and menopause, and the efficacy of treatment [10]. Elderly patients and those with multimorbidity also need to be studied to combat the rising prevalence of CVD risk factors such as hypertension, diabetes and obesity [11, 12]. Second, clinical care data contain a large number of individuals and wide range of clinical measurements, a combination that is difficult to obtain within a research setting. This facilitates the development of prediction models and decision support tools using artificial intelligence methods that can subsequently be implemented within the healthcare system. These tools can help healthcare professionals to interpret large amounts of patient data and assist healthcare decision-making. Third, researchers can use clinical care data to evaluate the current state of clinical practice, adherence to guidelines and develop treatment and referral strategies that better suit the current presentation of patients suspected of CVD.

However, data from earlier stages in the clinical care pathway remain difficult to access due to the smaller size of single general practitioner (GP) offices and the diversity of data collection systems. To close this gap, a collaboration was set up between the University Medical Center Utrecht (UMCU) and Cardiology Centers of the Netherlands (CCN), an organisation of 13 cardiac outpatient clinics that operate between the GP and the hospital cardiologist. In the Netherlands, CCN introduced “one-stop shop” cardiac outpatient clinics to facilitate efficient diagnostic workup for cardiac disease and fast diagnosis of potential life-threatening pathologies. GPs can refer their patients to a CCN clinic for cardiac workup when they suspect their patient suffers from cardiac disease. All CCN clinics perform the same standardised protocol and store their data in a shared data collection system. Follow-up appointments and results from referrals for advanced cardiac imaging or cardiac interventions are stored in the same system. As a result of this set-up, CCN offers a unique opportunity to obtain semi-structured data on a large group of patients at an early stage of the regular care pathway.

The aim of this paper is to describe the CCN clinical care database. The database contains data on a large number of individual patients and a wide range of standardised characteristics from a unique population situated between the GP and the hospital cardiologist. The clinical nature of the database ensures that it reflects the patient population currently seen in daily care, including those that may be underrepresented in clinical research. The database can be used to describe current clinical practice, evaluate the prevalence of cardiovascular risk factors and their relation to cardiovascular disease, and develop prediction algorithms that have the potential to be implemented in daily care.

## Construction and content

### Data generation at CCN clinics

#### Baseline examination

Every patient referred to one of the CCN clinics underwent a standardised diagnostic workup. This protocol consisted of transthoracic echocardiography (TTE) and ultrasound imaging of the carotid arteries, electrocardiography at rest (ECG) and during exercise (stress ECG), a laboratory test, and a consult with a nurse during which self-reported anthropometrics, symptoms, cardiovascular risk factors and comorbidities were registered. Past medication use and cardiovascular history were also recorded, as well as on site clinical diagnoses made by the cardiologist. An overview of all the stored clinical characteristics can be found in Table 1.

**Table 1:** Overview of all features stored in the database

Phase	Measurement
Baseline (2007-Feb 2018)	*Consult* (-) Presence and characteristics of cardiac symptoms (chest pain, dyspnoea, fatigue, palpitations, collapse, heart murmurs) (-) Anthropometrics (height, weight, hip circumference, blood pressure, heart rate, heart and breathing sounds, pulse, palpation) *Intake* (-) Behavioural cardiovascular risk factors (smoking, alcohol use) (-) Comorbidities (diabetes mellitus, hypertension, dyslipidaemia) (-) Family history of cardiovascular disease (atherosclerosis, sudden death, cardiomyopathy, arrhythmia) *Lab* (-) Lipids (total, high density, and low density cholesterol, triglycerides) (-) Potassium, sodium, haemoglobin, glucose (-) Glomerular filtration rate (-) Lipoprotein A, brain natriuretic protein, thyroid stimulation hormone *TTE* (-) M-mode (dimensions of aorta and left heart chambers) (-) Two-dimensional (evaluation of function and shape of all heart chambers and valves) (-) Colour Doppler (valve insufficiencies and septum defects) (-) Spectral Doppler (left ventricular diastolic function and gradients over valves) (-) Intima Media Thickness (left and right, anterior and posterior) *ECG* (-) Duration of defined ECG intervals and complexes (RR, PR, QRS, QT) (-) ST depression, elevation, negative T-top, QRS axis (-) Dilatation of left and right atrium, intraventricular conduction delay, left ventricular hypertrophy *Stress ECG* (-) Protocol, device, target heart rate, use of β-blocker before exercise test (-) ECG characteristics, blood pressure and heart rate before and during exercise test (-) Duration and load of exercise test, exercise tolerance, reason to stop exercise test (-) Arrhythmia or angina symptoms during exercise test, left ventricular hypertrophy *Decursus* (-) Cardiologist summary of visit (free text) *Medication* (-) Cardiovascular medication use grouped by researchers (-) Date medication was started and date it was ended when applicable *Diagnosis* (-) Cardiovascular diagnosis defined by researchers (-) Cardiovascular risk factor diagnosis defined by researchers (-) Date of diagnosis
Follow-up (2007—Feb 2018)	*Consult*, *Intake, Lab, TTE, ECG, Stress ECG* and *Decursus* as described for baseline *External procedures* (-) External procedure performed and location where it was performed (-) External procedure grouped by researchers (-) Date of appointment
Record linkage (2019)	All-cause mortality Educational level Ethnicity Personal income Cause-specific mortality

Body mass index was calculated based on self-reported height and weight. Blood pressure was measured with a Microlife WatchBP. TTE was performed with a General Electric Vivid E6 or E7 echocardiography device. Blood samples were analysed with the Roche Reflotron Sprint system. The ECG was recorded with the Welch Allyn Cardioperfect Pro recorder in supine position with 12 leads. The stress ECG was performed on a watt bike from Lode Corival Eccentric with simultaneous blood pressure measurements (Medtronic BL-6 Compact) and ECG recording (Welch Allyn Cardioperfect recorder). Raw data of the ECG, stress ECG and TTE were not available. Medication and diagnoses were recorded as semi-structured text.

**Fig. 1:**
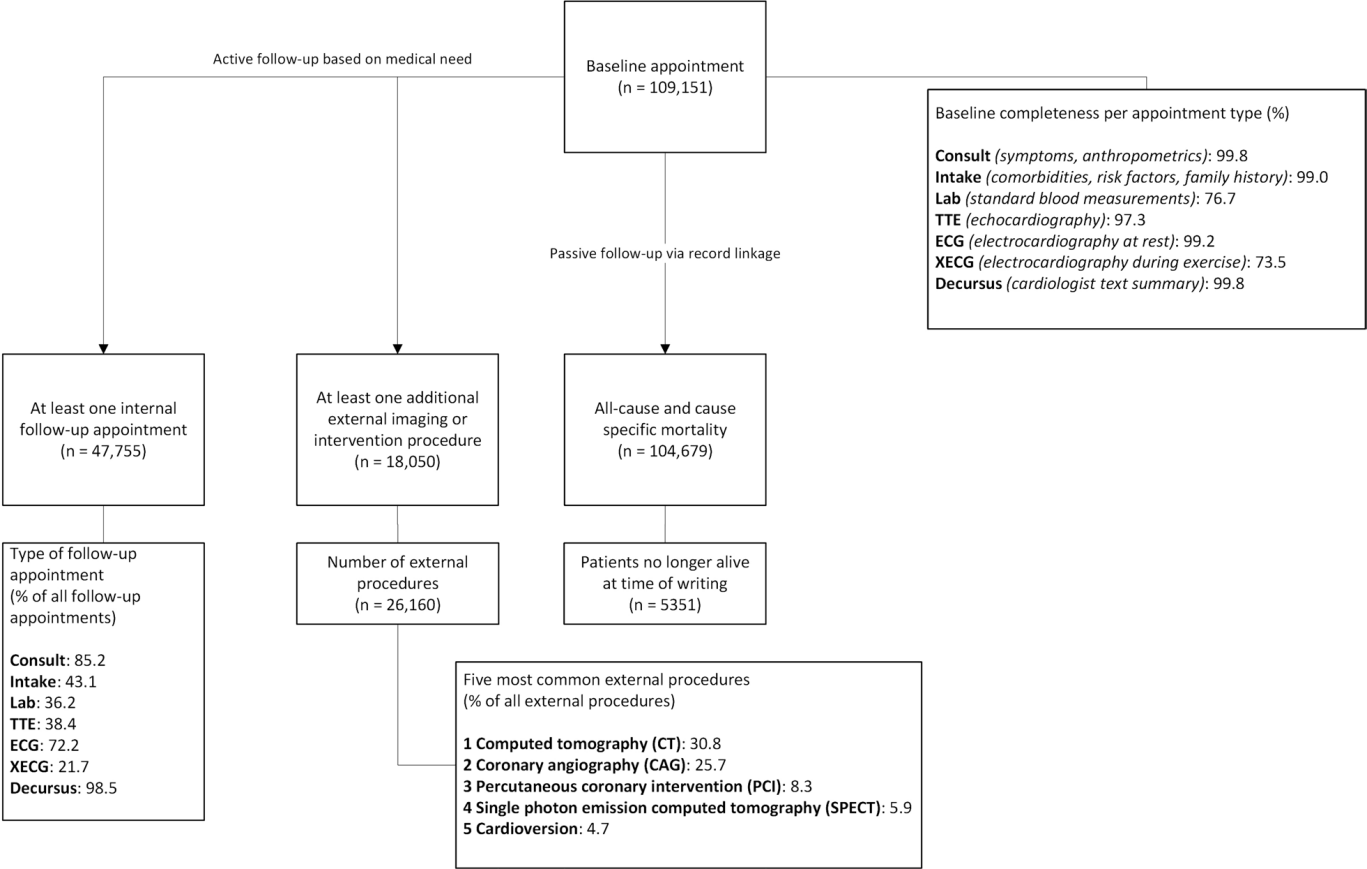
Overview of patient flow and completeness of measurements in the CCN database

While CCN has standardised and uniform diagnostic workup protocols for every patient, in practice a cardiologist may deviate from this protocol when this is clinically indicated. For example, the cardiologist may choose not to perform a stress ECG in patients with a contra-indication to the procedure, such as very high systolic blood pressure [13]. This introduces missing data, illustrated by the baseline stress ECG data which were missing for 25% of patients in the CCN database (Fig. 1).

#### Information collected during a patient’s clinical trajectory within CCN

After the first visit, patients may enter a clinical trajectory during which one or more return visits to a CCN clinic are planned. Information collected during these clinical follow-up visits was also stored in the CCN database. This clinical follow-up was not standardised but rather based on medical need. As a result, clinical follow-up varies across patients in frequency, duration, and measurements obtained. During these clinical follow-up visits either all or some components of the standard screening protocol were repeated, with rest ECG being repeated most frequently (Fig. 1).

Patients in need of additional imaging or cardiac intervention based on the result of their initial CCN workup were referred to a nearby hospital as these facilities were not available at the CCN clinics. The referral itself and the summarised text results of these procedures were stored in the CCN database (Table 1). Computed Tomography (CT) scans were performed most often, comprising 30.8% of all external procedures. The five most common external procedures can be found in Fig. 1.

### Database construction

#### Data extraction, cleaning and storage

We extracted all data generated by CCN up to February 2018 from their data collection system. These raw files were cleaned and processed using SAS (SAS Institute Inc., North Carolina, USA) to create a relational database. This process included separating first visit (baseline) data from follow-up visits, filtering out duplicated or empty entries and removing completely empty variables, streamlining variable names, and organising the data by type of clinical measurement (e.g. combine all laboratory measurements in one data table), among others. Raw unstructured text fields were checked for personal information, which was subsequently either removed while keeping the text field intact or the information was recoded into a new variable that no longer contained the personal information.

Raw medication use and diagnosis text data were structured into binary variables using text retrieval methods in R (R Core Team, Vienna, Austria). Medication entries were grouped into 23 categories of relevant cardiovascular medications based on either the brand name or the generic name, depending on which one was available (Additional file 1: Table S1). Diagnoses were divided into (i) Cardiovascular disease and (ii) Conditions that are risk factors for cardiovascular disease. The first category was subdivided into 5 subgroups, the second one into 4 subgroups (Additional file 1: Table S2).

The raw data and the clean relational database are stored within the UMCU infrastructure. The raw data is not available for researchers due to privacy constraints and is kept by the data manager. The anonymised versions of raw unstructured text fields are available, including raw medication and diagnosis data. Researchers can contact the authors for collaboration and access to the UMCU infrastructure. When the collaboration and the research topic have been agreed upon, external collaborators can get access to both the CCN database and all services and programmes supported by the UMCU. This includes artificial intelligence and advanced statistical programs. All work within the UMCU infrastructure will be stored, including analysis scripts and results. Access to the UMCU infrastructure will be retracted after the project has finished.

#### Passive and active follow-up outside the clinical trajectory

The CCN database has been linked to the national database of Statistics Netherlands for passive follow-up for all-cause and cause-specific mortality, and enrichment of the dataset with demographic and socioeconomic data. Linkage was successful for 95.9% of the database (Fig. 1). Failure to link likely occurred because a patient moved between their CCN visit and the moment of linking, as postal code was one of the linking factors. Linking of the CCN database with Statistics Netherlands was deemed appropriate by the ethical committee of Statistics Netherlands as it was in line with the CCN project aims.

Access to the following data was requested and granted: (i) all-cause and cause-specific mortality, (ii) education level and personal income and (iii) Personal Records Database, which among others contains information on country of birth. Access to the Personal Records Database also enables researchers to obtain a matched sample of the general population for comparison with the CCN population. In the future, the CCN database will be linked to other registries, such as the national hospitalisation registry, to obtain information on a more diverse set of outcome measures.

Patients could not be contacted for additional baseline questionnaires or active follow-up due to the pseudonymised nature of the database.

#### Missing data

Diagnostic procedures, treatments and follow-up of the patients were performed at the discretion of the treating cardiologist and thus driven by medical indication. This results in missing data for both baseline and follow-up visits. For example, more advanced biomarkers such as brain natriuretic peptide or high-sensitivity troponin will only be measured if the cardiologist suspects serious cardiac problems. Similarly, patients without entries in the medication or diagnosis file can be assumed to not use medication or be free of disease. Imputation strategies can be applied to deal with the missing values, but the preferred strategy depends on whether the data is likely to be missing at random or not. Researchers should be aware of the assumptions they make and describe these in their method section.

### Patient privacy

The CCN data were made available under implied consent and transferred to the UMCU under the Dutch Personal Data Protection Act. Patients were assigned a unique patient number that cannot be traced back to an individual without access to the original CCN data system, which is not available to UMCU researchers. This results in a pseudonymised database. The Medical Research Ethics Committee of the UMCU declared that the Medical Research Involving Human Subjects does not apply to this study. Unstructured text fields containing personal information were anonymised using an anonymization programme [14] before being included in the final research database.

### Content: describing the CCN study population

The CCN database contains data from 109,227 patients referred to one of the CCN clinics between February 2007 and February 2018 (Additional file 1: Fig. S1). Patients with missing data on age or sex or without records of their CCN visit were excluded (n = 76), bringing the total to 109,151 individuals with a mean age of 56 (± 15) years, of which 51.9% were women. About a third of the patients were 65 years or older and 12% had two or more comorbidities. Patients had a mean body mass index of 27.4 (± 20) kg/m^2^ and an average systolic blood pressure of 141 (± 22) mmHg. The majority of patients had a positive cardiovascular family history (65.2%) and 14.9% of patients suffered from cardiovascular disease at baseline. Approximately one third of patients were current smokers (36.8%), 29.7% had hypertension, 15.6% had dyslipidaemia and 8% had diabetes mellitus (DM) (Table 2).

**Table 2:** Baseline characteristics of patients in the CCN database

Variable	Whole database n = 109,151	Women n = 56,628	Men n = 52,524	Missing data (%)
General				
Women (n, %)	56,628 (51.9)			
Age (years)	56 (15)	57 (15)	56 (15)	
Age categories (n, %)				
Under 50	33,165 (30.4)	16,954 (29.9)	16,211 (30.9)	
50–64	41,273 (37.8)	20,859 (36.8)	20,414 (38.9)	
65–74	22,931 (21.0)	12,152 (21.5)	10,779 (20.5)	
75 and older	11,781 (10.8)	6662 (11.8)	5119 (9.7)	
Body mass index (kg/m^2^)	27.4 (20.0)	27.3 (20.2)	27.5 (19.8)	2.9
Systolic blood pressure (mmHg)	141 (22)	140 (23)	143 (20)	2.9
Current smoker (n,%)	40,139 (36.8)	20,712 (36.6)	19,427 (37)	8.9
Ever smoker (n,%)	71,659 (65.7)	35,508 (62.7)	36,151 (68.8)	8.8
Cardiovascular disease (CVD) (n, %)				
History of CVD^a^	16,311 (14.9)	6,483 (11.4)	9,828 (18.7)	
Family history of CVD^b^	71,148 (65.2)	39,318 (69.4)	31,830 (60.6)	17.8
History of other cardiovascular conditions^c^	23,957 (21.9)	11,804 (20.8)	12,153 (23.1)	
Comorbidities (n, %)				
Hypertension	32,460 (29.7)	17,290 (30.5)	15,270 (28.9)	2.5
Dyslipidaemia	16,978 (15.6)	8148 (14.4)	8830 (16.8)	2.5
Diabetes mellitus	8709 (8.0)	3967 (7.0)	4742 (9.0)	2.6
Number of comorbidities				
0	64,199 (58.8)	33,799 (59.9)	30,400 (57.9)	
1	28,705 (26.3)	15,081 (26.6)	13,624 (25.9)	
2	11,001 (10.1)	5392 (9.5)	5609 (10.7)	
3	2382 (2.2)	1125 (2.0)	1257 (2.4)	

All values are given as mean (SD) unless otherwise specified

^a^History of CVD = diagnosis of heart failure, coronary heart disease, cerebrovascular disease or congenital heart disease before baseline appointment, or invasive cardiac intervention

^b^Family history of CVD = family history of atherosclerosis, sudden death, cardiomyopathy or arrhythmia

^c^History of other cardiovascular conditions = diagnosis of arrhythmia, valvular disease, cardiomyopathy, atherosclerosis, peripheral artery disease or abdominal aneurysm before baseline appointment, or non-invasive cardiac or peripheral intervention

The majority of patients (56.1%, n = 61,232) only had a baseline visit, 17.5% (n = 19,111) had one follow-up visit at CCN, and 26.3% (n = 28,808) had three or more follow-up visits at CCN. Compared with patients who were seen once, those with at least one clinical follow-up appointment were older at baseline (60 vs 54 years), had a higher systolic blood pressure (145 vs 138 mmHg) and were more often current smokers (41.1 vs 33.4%). In addition, they more often had a history of cardiovascular disease (20.6 vs 10.5%), prevalent cardiovascular risk conditions (30.0 vs 15.5%), and comorbidities (Additional file 1: Table S3).

In total, 18,050 (16.5%) patients were referred for an external procedure (Fig. 1). Compared with patients who were not referred, patients with at least one external procedure were older at baseline (60 vs 56 years) and had a higher prevalence of comorbidities and CVD history (21.3% vs 13.7%). Women were less often referred for an external procedure (46.1 vs 53.0%) (Additional file 1: Table S4).

The CCN database consists of data derived from medical care and thus participants were not actively recruited, nor were there explicit in- and exclusion criteria. Data on patients who were not referred to CCN are not available, so we were unable to compare patients referred to CCN with those who were not. However, to approximate this comparison, we compared the socioeconomic characteristics of the CCN database to an age- and sex-matched sample of the general population. Patients referred to CCN were more often of Dutch descent (77.2% vs 70.8%) and had a higher median annual personal income (€27,914 vs €22,270) than the general population (Table 3).

**Table 3:** Sociodemographic characteristics of the CCN database and a sample of the general population matched on year of birth and sex

	CCN database (n = 104,519)^a^	General population (n = 104,519)
Origin^b^ (n, %)		
Native Dutch	80,692 (77.2)	74,042 (70.8)
First generation immigrant	15,731 (15.1)	24,592 (23.5)
Second generation immigrant	8096 (7.7)	5884 (5.6)
Annual personal income (€)	27,914 [14,822–47,344]	22,270 [11,900–38,758]
Annual personal income groups (n, %)		
Negative or zero	4760 (4.6)	5701 (5.5)
< €20.000	33,209 (31.8)	33,048 (31.6)
€20.000—€50.000	42,325 (40.5)	33,906 (32.4)
€50.000—€100.000	18,204 (17.4)	10,530 (10.1)
€100.000—€200.000	4197 (4.0)	1675 (1.6)
≥ €200.000	1121 (1.1)	337 (0.3)
Not available	703 (0.7)	19,321 (18.5)

Values are given as median (IQR) unless otherwise specified

^a^Year of birth could not be re-calculated for 160 study participants, so these could not be matched with the general population and are thus removed from this table

^b^Origin was defined as (i) Native Dutch; both parents born in the Netherlands, (ii) First generation immigrant; person born outside the Netherlands with at least one parent born outside the Netherlands, (iii) Second generation immigrant; person born in the Netherlands with at least one parent born outside the Netherlands

## Utility and discussion

### Utility: intended use and database benefits

The main strength of the CCN database lies in its combination of a large study population and a large number of different, and sometimes longitudinal, measurements per individual. Such data is difficult to obtain in cohorts specifically set up for research as funds are often not sufficient to cover both including a large population and collecting a large number of (longitudinal) measurements. In addition, the CCN database captures a unique population situated between the GP and the hospital that is rarely seen in clinical studies.

Clinical care databases like the CCN database can make important contributions to three areas of research due to some of their inherent characteristics. First, these databases reflect the population currently seen in clinical care and thus include groups that are traditionally underrepresented in research [8]. We show that women comprise 52% of the CCN database, providing a valuable foundation for research into both differences between the sexes and women-specific cardiovascular disease presentations and risk factors [15, 16]. Similarly, the CCN dataset contains 11,781 patients aged 75 years and older and 13,383 patients with two or more comorbidities, offering researchers an opportunity to verify if study outcomes also apply to these patient groups. These numbers illustrate the potential value of the CCN database for addressing research questions about underrepresented patient groups that have remained unanswered due to scarcity of data.

Second, the size of clinical care databases that combine a large study population with a large number of measurements per individual creates opportunities for the application of artificial intelligence methods. The CCN database contains more than 300 informative features on over 100.000 patients that can be used for the development of artificial intelligence-based prediction algorithms and decision support tools. In addition, the CCN database contains several anonymised Dutch free text fields, which can be used for the development of text analysis algorithms specific for Dutch clinical notes. This is an important area of research, as many existing text analysis resources are based on English clinical text [17]. These programmes can subsequently be used to extract and structure valuable information from free text and turn it into a usable format for researchers.

Third, clinical care databases reflect medical practice allowing for comparisons between clinical care and the recommendations in the prevailing guidelines. Such perspectives spark debate on inconsistencies that may exist between guidelines and current practice. The CCN database functions in this case as a tool to bridge the gap between guidelines based mainly on clinical research and the reality of daily cardiac care.

### Discussion: comparison of performance and functionality with similar existing databases

However, the CCN database also has some limitations that need to be addressed. We will discuss the two main ones, data quality and generalisability.

#### Data quality: missing data and measurement errors

The data within the CCN database was collected for care purposes and not for research. As a result, data collection and follow-up during the medical trajectory are not uniform across patients. Similarly, the database may not contain all clinical information researchers need, such as highly specific biomarkers, because these are not normally collected in daily care. Furthermore, raw ECG data and echocardiographic images were saved to a different system than the standardised clinical data and were thus not stored in the CCN database. These limitations are in part inherent to the database, so researchers should consider whether the CCN database is ‘fit for purpose’ for their specific research question. However, some of these limitations can be addressed and alleviated. To obtain standardised follow-up for all individuals in the CCN database, we performed record linkage for all-cause and cause-specific mortality. We plan to include follow-up for non-fatal outcomes in the future, as these outcomes are clinically relevant for the relatively young and healthy CCN population. To alleviate the issue of missing data on important confounders such as socioeconomic status, we enriched the CCN database with information on ethnicity, educational level and personal income through record linkage. Text mining approaches can be used to further enrich the CCN database if the required information can be found within the unstructured text fields. Available missing data techniques such as multiple imputation can be used to address remaining missing values as long as researchers carefully consider the assumptions underlying these techniques.

Data collection and entry in the CCN database is not checked as vigorously as in databases created for research, so data entry mistakes and slightly differential measurement practices across CCN clinics may introduce measurement error and misclassification. We have tried to correct the most obvious data entry errors to reduce its effect, but researchers should consider the possibility of differential measurement error and the resulting risk of misclassification bias when interpreting their results.

#### Generalisability and comparison to other databases

The CCN database is comprised of patients who were referred by their GP on suspicion of cardiac disease. We were unable to compare those included in the CCN database with those who were not referred, but we were able to approach this comparison by using an age- and sex-matched sample from the general population. We show that CCN patients have a higher socio-economic status and are more often native Dutch compared with the general population. Moreover, the prevalence of DM in the CCN database seems to be similar to that in the Netherlands as a whole [18], while we expected a higher prevalence given that CCN screens patients at elevated cardiovascular disease risk. However, GPs may refer DM patients with cardiac complaints to a DM-specific outpatient clinic instead of a CCN clinic, resulting in a low DM prevalence within the CCN database. This suggests there is some selection bias occurring within the clinical care pathway, where relatively healthy Dutch patients with higher socio-economic status are more often referred to a CCN clinic than those with lower socio-economic status or those of non-Dutch descent.

There are examples of other clinical care databases such as the hospital-based UPOD database [19] and the Julius General Practitioner’s Network [20]. However, these include distinctively different patient populations, as the first collects data from within the hospital and the second from within GP practice. The CCN database is unique in that it captures the patients in between these two.

## Conclusion

The CCN database is a regular care database containing data from 109.151 patients collected between 2007 and 2018. This database offers the opportunity to perform research in a unique study population that reflects the patient population seen in daily cardiology practice, including women, the elderly, and patients with multiple comorbidities. The size of this database facilitates the application of artificial intelligence methods. Moreover, the features in the database make it possible to describe current cardiology practice and evaluate this against guidelines based primarily on results from clinical trials.

## Supplementary Information

**Additional file 1.** Supplementary figure and tables.

## Data Availability

The CCN database are not publicly available due to ethical and data protection constraints, but are available from the corresponding author on reasonable request. Proposals for possible collaborations should be addressed to Dr Leonard Hofstra (L.Hofstra@cardiologiecentra.nl) or Dr Hester den Ruijter (H.M.denRuijter-2@umcutrecht.nl).

## Abbreviations

CCN: Cardiology Centers of the Netherlands
CT: Computed Tomography
CVD: Cardiovascular disease
DM: Diabetes Mellitus
ECG: Electrocardiography
GP: General practitioner
TTE: Transthoracic echocardiography
UMCU: University Medical Center Utrecht
Stress ECG: Stress-electrocardiography
