# Supplementary material for: Routine clinical care data from thirteen cardiac outpatient clinics: design of the Cardiology Centers of the Netherlands (CCN) database
Source: BMC Cardiovasc Disord. 2021 Jun 10;21:287. doi: 10.1186/s12872-021-02020-7 (PMC8191101; doi:10.1186/s12872-021-02020-7)
Supplement: Supplementary file 1 — Additional file 1. Supplementary figure and tables. [file 12872_2021_2020_MOESM1_ESM.docx]

# Additional File 1

## Figures

Figure S1 All patients included in the CCN database throughout the Netherlands


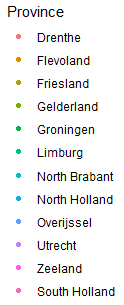


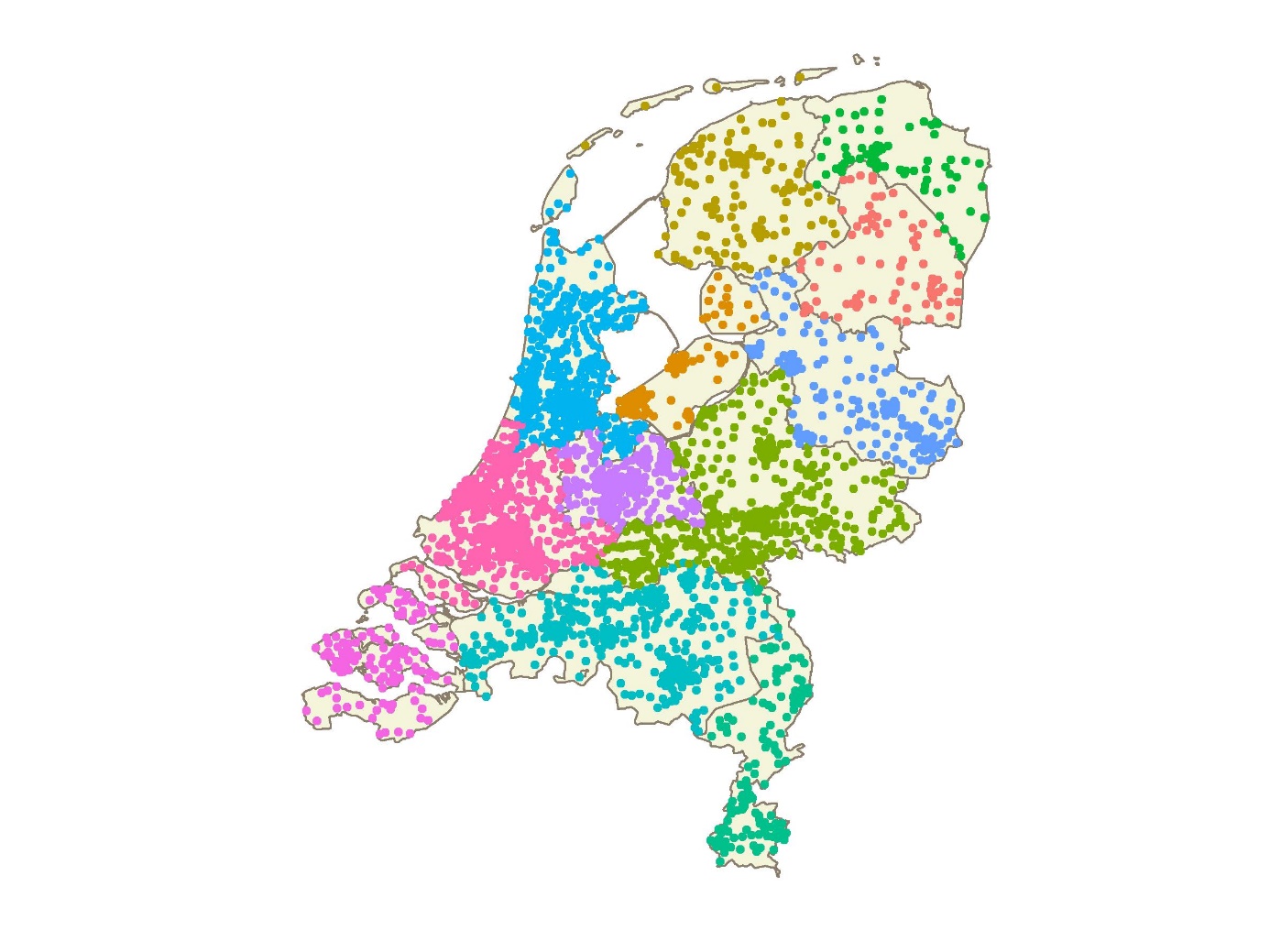


## Tables

| **Group** | **Medications included** |
| --- | --- |
| Aspirin | Acetylsalicic acid, Carbasalate calcium |
| Angiotensin-converting-enzyme inhibitor (ACEI) | Benazepril, Perindopril, Captopril, Cilazapril, Delapril, Enalapril, Fosinopril, Lisinopril, Quinapril, Ramipril, Trandolapril, Zofenopril |
| Angiotensin receptor blocker (ARB) | Candesartan, Eprosartan, Irbesartan, Losartan, Olmesartan, Telmisartan, Valsartan |
| Thiazides | Hydrochlorothiazide, Chlorthalidone, Indapamide |
| Potassium-sparing diuretics | Eplerenone, Spironolactone, Triamterene |
| Loop diuretics | Bumetanide, Furosemide |
| Beta-blocker | Acebutolol, Atenolol, Bisoprolol, Carvedilol, Celiprolol, Labetalol, Metoprolol, Nebivolol, Pindolol, Propranolol, Sotalol |
| Calcium-channel blocker | Amlodipine, Barnidipine, Felodipine, Isradipine, , Lacidipine, Lercanidipine, Nicardipine, Nifedipine, Nimodipine, Nitrendipine, Diltiazem, Verapamil |
| Alpha-blocker | Alfuzosine, Doxazosine, Silodosine, Tamsulosine, Terazosine, Urapidil |
| Nitrates | Isosorbide dinitrate, , Isosorbide mononitrate, Nicorandil, Nitroglycerine |
| Digoxin | Digoxin |
| Statins | Atorvastatine, Fluvastatine, Pitavastatine, Pravastatine, Rosuvastatine, Simvastatine |
| Metformin | Metformin |
| Insulin | Insuline |
| Ezetimibe | Ezetimibe |
| Sulphonylureas | Glibenclamide, Glimepiride, Tolbutamide, Gliclazide |
| Fibrates | Bezafibrate, Ciprofibrate, Gemfibrozil, Fenofibrate |
| P2Y12-Inhibitor | Prasugrel, Clopidogrel, Ticagrelor |
| Dipyridamole | Dipyridamole |
| Ivabradine | Ivabradine |
| Non Vitamin-K oral anticoagulant (NOAC) | Apixaban, Edoxaban, Rivaroxaban, Dabigatran |
| Anti-arrhythmics | Amiodarone, Disopyramide, Flecainide, Kinidine, Lidocaine  Propafenone |
| Vitamin-K Antagonist | Acenocoumarol, Phenprocoumon |
| Other | Any medication that is not in any of the groups described above |

Table S1 Medication names per medication group

| **Group** | **Diagnoses included** |
| --- | --- |
| Cardiovascular disease | |
| Heart failure | Left ventricular hypertrophy, left ventricular dysfunction, concentric hypertrophic left ventricle, concentric left ventricle, decompensatio cordis, heart failure, diastolic dysfunction, coronary microvascular disease, poor ventricular function |
| Coronary heart disease | Myocardial infarction, angina pectoris, anginal symptoms, chest pain, acute coronary syndrome, silent ischaemia, coronary disease, heart revalidation, coronary insufficiency, 1/2/3 artery disease |
| Cerebrovascular disease | Cerebrovascular accident, transient ischaemic attack, subarachnoid haemorrhage, eye infarct, brain infarct, brain bleeding, stroke, subarachnoidal bleeding, cerebral infarct, cerebrovascular infarct, retina infarct, lacunar infarct |
| Congenital heart disease | Tetralogy of Fallot, ventricular septum defect, atrial septum defect, septum defect, coarctatio aortae, foramen ovale, Ductus Botalli |
| Cardiovascular intervention | Percutaneous coronary intervention, stent, coronary artery bypass graft, bypass, revascularisation, grafting, dotter, percutaneous transluminal coronary angioplasty, valve replacement, valvuloplasty, transcatheter aortic valve implantation, aortic valve replacement, mitral valve replacement, commissurotomy, myocardial perfusion scan, heart catheterisation, implementation of pacemaker or implantable cardioverter-defibrillator |
| Conditions that are risk factors for cardiovascular disease | |
| Other cardiovascular disease | Cardiomyopathy, atherosclerosis, abdominal aortic aneurysm, peripheral vascular disease, arteriosclerosis, claudicatio intermittens, deep vein thrombosis, venous thrombosis, venous insufficiency, phlebitis |
| Arrhythmia | Atrial fibrillation, ventricular fibrillation, atrium flutter, ventricular flutter, paroxysmal atrial fibrillation, conduction delay, supraventricular tachycardia, sick sinus syndrome, sinus exit block, Wolff-Parkinson-White, atrioventricular nodal re-entry tachycardia, extrasystoles, ventricular extrasystoles, arrhythmia, bradycardia, tachycardia, bigemini, AV block, right bundle branch block, left bundle branch block, left anterior hemiblock, premature ventricular contractions, premature atrial contractions, atrial extrasystoles, hemiblock, rhythm disorder |
| Valvular disease | Valve stenosis, valve sclerosis, valve insufficiency, regurgitation, mitral insufficiency, tricuspid insufficiency, valve defect, mitral regurgitation, mitral stenosis, valve disease |
| Risk factor intervention | Ablation, radiofrequency catheter ablation, cardioversion, electrocardioversion, percutaneous transluminal angioplasty, endarterectomy, aortic bifurcation prosthesis, abdominal aortic stent |

Table S2 Diagnoses per diagnosis group

| **Variable** | **Whole cohort**  n = 109,151 | **Follow-up**  n = 47,755 | **No follow-up**  n = 61,396 | **Missing data (%)** |
| --- | --- | --- | --- | --- |
| **General** | | | | |
| Women (n,%) | 56,628 (51.9) | 24,271 (50.8) | 32,357 (52.7) |  |
| Age (years) | 56 (15) | 60 (14) | 54 (16) |  |
| Body mass index (kg/m^2^) | 27.4 (20.0) | 27.8 (24.4) | 27.0 (15.9) | 2.9 |
| Systolic blood pressure (mmHg) | 141 (22) | 145 (22) | 138 (20) | 2.9 |
| Current smoker (n,%) | 40,139 (36.8) | 19.645 (41.1) | 20,494 (33.4) | 8.9 |
| Ever smoker (n,%) | 71,659 (65.7) | 34,250 (71.7) | 37,409 (60.9) | 8.8 |
|  |  |  |  |  |
| **Cardiovascular disease (CVD)** | | | | |
| History of CVD (n,%) | 16,311 (14.9) | 9,845 (20.6) | 6,466 (10.5) |  |
| Family history of CVD (n,%) | 71,148 (65.2) | 31,125 (65.2) | 40,023 (65.2) | 17.8 |
| CVD risk factor conditions (n,%) | 23,957 (21.9) | 14,465 (30.3) | 9,492 (15.5) |  |
|  |  |  |  |  |
| **Comorbidities** | | | | |
| Hypertension (n,%) | 32,460 (29.7) | 17,238 (36.1) | 15,222 (24.8) | 2.5 |
| Dyslipidaemia (n,%) | 16,978 (15.6) | 8,765 (18.4) | 8,213 (13.4) | 2.5 |
| Diabetes mellitus (n,%) | 8,709 (8.0) | 4,329 (9.1) | 4,380 (7.1) | 2.6 |

Table S3 Baseline characteristics stratified by follow-up status

| **Variable** | **Whole cohort**  n = 109,151 | **External procedure**  n = 18,050 | **No external procedure**  n = 91,101 | **Missing data (%)** |
| --- | --- | --- | --- | --- |
| **General** | | | | |
| Women (n,%) | 56,628 (51.9) | 8,322 (46.1) | 48,306 (53.0) |  |
| Age (years) | 56 (15) | 60 (12) | 56 (16) |  |
| Body mass index (kg/m^2^) | 27.4 (20.0) | 27.7 (15.3) | 27.3 (20.8) | 2.9 |
| Systolic blood pressure (mmHg) | 141 (22) | 144 (21) | 141 (22) | 2.9 |
| Current smoker (n,%) | 40,139 (36.8) | 6,169 (34.2) | 33,970 (37.3) | 8.9 |
| Ever smoker (n,%) | 71,659 (65.7) | 11,981 (66.4) | 59,678 (65.5) | 8.8 |
|  |  |  |  |  |
| **Cardiovascular disease (CVD)** | | | | |
| History of CVD (n,%) | 16,311 (14.9) | 3,839 (21.3) | 12,472 (13.7) |  |
| Family history of CVD (n,%) | 71,148 (65.2) | 12,492 (69.2) | 58,656 (64.4) | 17.8 |
| CVD risk factor conditions (n,%) | 23,957 (21.9) | 4,531 (25.1) | 19,426 (21.3) |  |
|  |  |  |  |  |
| **Comorbidities** | | | | |
| Hypertension (n,%) | 32,460 (29.7) | 6,389 (35.4) | 26,071 (28.6) | 2.5 |
| Dyslipidaemia (n,%) | 16,978 (15.6) | 3,583 (19.9) | 13,395 (14.7) | 2.5 |
| Diabetes mellitus (n,%) | 8,709 (8.0) | 1,864 (10.3) | 6,845 (7.5) | 2.6 |

Table S4 Baseline characteristics stratified by external referral status
